# Population-Based Long-Term Cardiac-Specific Mortality Among 34 489 Five-Year Survivors of Childhood Cancer in Great Britain

**DOI:** 10.1161/CIRCULATIONAHA.116.024811

**Published:** 2017-03-06

**Authors:** Miranda M. Fidler, Raoul C. Reulen, Katherine Henson, Julie Kelly, David Cutter, Gill A. Levitt, Clare Frobisher, David L. Winter, Michael M. Hawkins

**Affiliations:** From Centre for Childhood Cancer Survivor Studies, Institute of Applied Health Research, University of Birmingham, Birmingham, UK (M.M.F., R.C.R., K.H., J.K., C.F., D.L.W., M.M.H.); Nuffield Department of Population Health, University of Oxford, Oxford, UK (K.H., D.C.); Oxford Cancer and Haematology Centre, Oxford University Hospitals NHS Foundation Trust, Churchill Hospital, Headington, Oxford, UK (D.C.); and Great Ormond Street Hospital NHS Foundation Trust, London, UK (G.A.L.).

**Keywords:** mortality, neoplasms, pediatrics

## Abstract

**Background:**

Increased risks of cardiac morbidity and mortality among childhood cancer survivors have been described previously. However, little is known about the very long-term risks of cardiac mortality and whether the risk has decreased among those more recently diagnosed. We investigated the risk of long-term cardiac mortality among survivors within the recently extended British Childhood Cancer Survivor Study.

**Methods:**

The British Childhood Cancer Survivor Study is a population-based cohort of 34 489 five-year survivors of childhood cancer diagnosed from 1940 to 2006 and followed up until February 28, 2014, and is the largest cohort to date to assess late cardiac mortality. Standardized mortality ratios and absolute excess risks were used to quantify cardiac mortality excess risk. Multivariable Poisson regression models were used to evaluate the simultaneous effect of risk factors. Likelihood ratio tests were used to test for heterogeneity and trends.

**Results:**

Overall, 181 cardiac deaths were observed, which was 3.4 times that expected. Survivors were 2.5 times and 5.9 times more at risk of ischemic heart disease and cardiomyopathy/heart failure death, respectively, than expected. Among those >60 years of age, subsequent primary neoplasms, cardiac disease, and other circulatory conditions accounted for 31%, 22%, and 15% of all excess deaths, respectively, providing clear focus for preventive interventions. The risk of both overall cardiac and cardiomyopathy/heart failure mortality was greatest among those diagnosed from 1980 to 1989. Specifically, for cardiomyopathy/heart failure deaths, survivors diagnosed from 1980 to 1989 had 28.9 times the excess number of deaths observed for survivors diagnosed either before 1970 or from 1990 on.

**Conclusions:**

Excess cardiac mortality among 5-year survivors of childhood cancer remains increased beyond 50 years of age and has clear messages in terms of prevention strategies. However, the fact that the risk was greatest in those diagnosed from 1980 to 1989 suggests that initiatives to reduce cardiotoxicity among those treated more recently may be having a measurable impact.

## Abstract 2

Supplemental Digital Content is available in the text.

Treatment for childhood cancer has changed substantially over the past 50 years. In particular, since 1990, there have been attempts to reduce treatment intensity among children with relatively good-prognosis neoplasms with the aim of reducing the risk of treatment-related morbidity and mortality. Previous research has identified that thoracic radiotherapy and specific types of chemotherapy, particularly anthracyclines, which were introduced in the late 1970s, increase the risk of cardiac disease among survivors of childhood cancer.^1,2^ Thus, it is important to assess whether there is evidence of a measurable decline in the risk of death from cardiac disease among those treated since 1990.

However, to date, only 2 studies have investigated the risk of cardiac mortality in relation to treatment era, both of which were restricted by narrow treatment era time spans.^3,4^ For this reason, we have investigated the risk of cardiac mortality among nearly 35 000 survivors of childhood cancer included in the BCCSS (British Childhood Cancer Survivor Study), the largest study to assess mortality in survivors of childhood cancer.^5^ Principal advantages of the BCCSS compared with previous studies are that it is large-scale and population based and includes survivors treated across almost 7 decades (1940–2006), enabling an assessment of the risk of cardiac mortality in relation to a wide span of treatment eras. Furthermore, because the BCCSS has an unrivaled number of survivors from earlier treatment decades, we have undertaken the most powerful investigation of excess cardiac mortality risks among childhood cancer survivors >50 years of age. As a result, the findings from this study provide new evidence with which to inform survivors of childhood cancer and clinicians and to update clinical follow-up guidelines.

## Methods

### The BCCSS

The BCCSS comprises 34 489 five-year survivors of childhood cancer diagnosed under 15 years age from 1940 to 2006 in Britain. The cohort was the first national population-based study of survivors of childhood cancer to be untaken in Great Britain and was identified from the National Registry of Childhood Tumors, which has an ≈99% ascertainment rate.^6^ The study is maintained at the Center for Childhood Cancer Survivor Studies, where research on a wide spectrum of possible adverse health outcomes of childhood cancer and its treatment is undertaken. Additional details relating to the study may be found online^7^ and in the descriptive article concerned with methodological aspects underlying the BCCSS.^8^ Ethics approval for the study was obtained from the National Research Ethics Service, and legal approval to process identifiable data without consent was approved by the Confidentiality Advisory Group. Descriptive characteristics of the BCCSS cohort can be found in Tables I–III in the online-only Data Supplement.

### Death Ascertainment

Collaboration with the Health and Social Care Information Center (England and Wales) and National Health Service Central Register (Scotland) enabled us to ascertain each survivor’s vital and residency/emigration status. For each death, an attempt was made to obtain the death certificate and underlying cause of death as coded by the Office of National Statistics (England and Wales) and National Records of Scotland (Scotland) using the relevant *International Classification of Diseases*. *International Classification of Diseases* codes corresponding to a cardiac death were identified and subcategorized into clinically relevant groups for analysis, specifically ischemic heart disease (IHD), cardiomyopathy/heart failure (CM/HF), arrhythmias, pericardial disease, and valvular disease (Table IV in the online-only Data Supplement). Follow-up for cardiac mortality began at 5-year survival and continued until the first instance of emigration, death, or February 28, 2014.

### Statistical Analyses

Standardized mortality ratios (SMRs) and absolute excess risks (AERs) were calculated with standard cohort techniques.^9^ The SMR was defined as the observed divided by the expected number of deaths. The AER was defined as the observed minus the expected number of deaths divided by person-years at risk multiplied by 10 000. Expected numbers were calculated by multiplying the person-years within each sex-, age- (quinquennial), and calendar year- (single year) specific stratum by the corresponding mortality rate for the population of England and Wales and then summing across the strata.^10^

Multivariable Poisson regression models for the SMRs and AERs were then fitted to evaluate the simultaneous effect of the following demographic- and cancer-related factors: sex, first primary neoplasm (FPN) type, age at cancer diagnosis, treatment era of diagnosis, and attained age. The results from the adjusted multivariable model were reported in terms of relative risks (which may be interpreted as the ratios of the SMRs adjusted for other factors fitted) and excess mortality ratios (EMRs; which may be interpreted as the ratios of the AERs adjusted for other factors fitted^9^; Table V in the online-only Data Supplement). To test for heterogeneity or linear trend, likelihood ratio tests within Poisson regression models were used. *P* for heterogeneity tests the null hypothesis that the relative risks or EMRs are equal across levels of the factor concerned, against the alternative hypothesis that the relative risks or EMRs vary across levels of the factor, having adjusted for other factors included within the model. *P* for trend tests the null hypothesis that the relative risks or EMRs are equal across levels of the factor concerned, against the alternative hypothesis that the relative risks or EMRs increase or decrease linearly across levels of the factor, having adjusted for other factors included within the model. Because we anticipated a priori that there might be evidence of risks increasing and then declining as treatment era became more recent, particularly in relation to anthracycline-related CM/HF, we also investigated for evidence of a quadratic relationship in risks with treatment era using likelihood ratio tests within multivariable Poisson regression models. *P* for quadratic tests whether there is evidence of such a specific type of systematic nonlinear variation in the relative risks or EMRs across levels of the factor concerned, having adjusted for other factors included within the model.

Cumulative mortality, as a function of follow-up, was estimated by use of the stcompet command in Stata.^11^ Causes of death other than the one under study were treated as competing risks.^12,13^ To test for heterogeneity among cumulative mortality curves, log-rank tests were used.

All analyses were completed with Stata 13.1,^11^ for which the criterion for statistical significance was a 2-sided value of *P*<0.05.

### Patient Involvement

Overall, survivors showed their support for the study by returning 10 488 BCCSS questionnaires, which represented 80% of those sent to survivors. Furthermore, almost all survivors completing the BCCSS questionnaire requested receipt of study newsletters, the means by which we inform survivors of the findings of the research. Last, 2 patient representatives attend the BCCSS Steering Group meetings and provide feedback on research priorities and research translation.

## Results

### Study Characteristics

From 5-year survival, the cohort was followed up for a total of 620 753 person-years, with a mean follow-up of 18.0 years (range, 0.0–68.7 years) and to a mean attained age of 29.6 years (range, 5.5–85.6 years). By the study exit date, 4475 individuals (13.0%) in the cohort had died; of those deaths, 181 (4.0%) were attributed to cardiac causes (Table 1). The mean follow-up time from diagnosis and attained age at cardiac death were 31.4 and 39.2 years, respectively, which were greater than that observed for individuals who died of a noncardiac cause. Men accounted for approximately two thirds (63.5%) of the cardiac deaths, and survivors of central nervous system tumors (excluding primitive neuroectodermal tumor), Hodgkin lymphoma (HL), and Wilms accounted for nearly 50% of the cardiac deaths observed when combined.

**Table 1.**
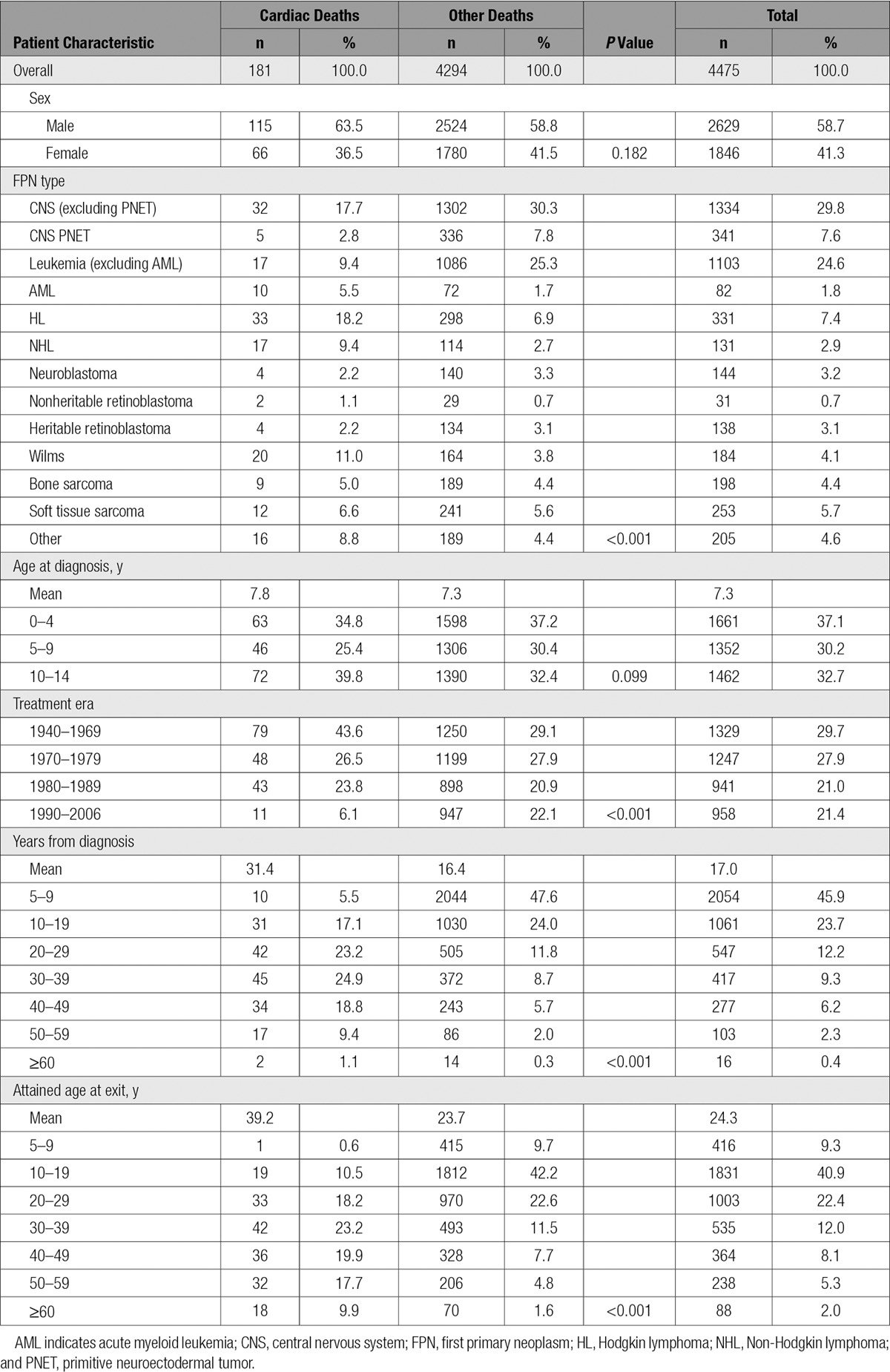
Study Characteristics of the British Childhood Cancer Survivor Study

### Overall Cardiac Mortality

Survivors of childhood cancer experienced 3.4 times (95% confidence interval [CI], 2.9–3.9) the number of cardiac deaths expected from the general population, which equated to 2.1 (95% CI, 1.6–2.5) excess cardiac deaths per 10 000 person-years (Table 2). All FPN types with at least 5 observed cardiac deaths were at a statistically significant increased risk of cardiac death (Table 3). The SMR was substantially raised (SMR>5.0) for survivors of acute myeloid leukemia, Wilms, and HL at 23.5 (95% CI, 11.2–43.1), 6.5 (95% CI, 4.0–10.0), and 5.4 (95% CI, 3.7–7.6), respectively. From 5 to 19 to >60 years of age, the SMR declined from 9.7-fold (95% CI, 5.9–15.0) to 2.2-fold (95% CI, 1.3–3.5) that expected, respectively, whereas for the same age groupings the AER rose from 0.7 (95% CI, 0.4–1.1) to 23.7 (95% CI, 3.6–43.8) excess cardiac deaths per 10 000 person-years; both of these trends were statistically significant (SMR *P* for trend=0.0065; AER *P* for trend<0.0001). When assessed by treatment era, evidence of a quadratic relationship was identified for the SMRs (*P* for quadratic=0.0014) and AERs (*P* for quadratic=0.0072), where after adjustment the risk was higher among those treated in the 1980s than those treated in decades before or since (Table 4). Compared with those diagnosed before 1970, the relative risk for cardiac death was 1.6 times (95% CI, 1.1–2.5), 2.3 times (95% CI, 1.4–3.8), and 1.0 times (95% CI, 0.4–2.1) higher among those diagnosed in 1970 to 1979, 1980 to 1989, and 1990 to 2006, respectively, after adjustment for sex, FPN type, age at diagnosis, and attained age.

**Table 2.**
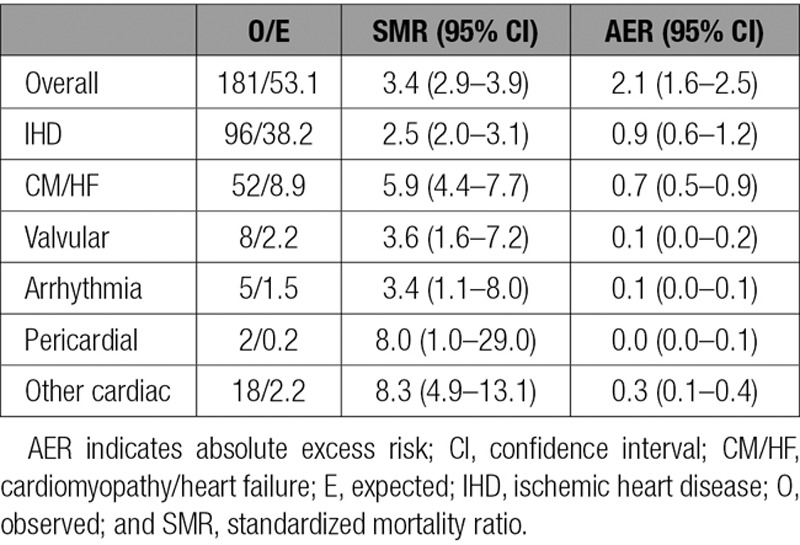
Standardized Mortality Ratios and Absolute Excess Risks per 10 000 Person-Years for Deaths Resulting From All Cardiac Causes and Cardiac-Specific Causes

**Table 3.**
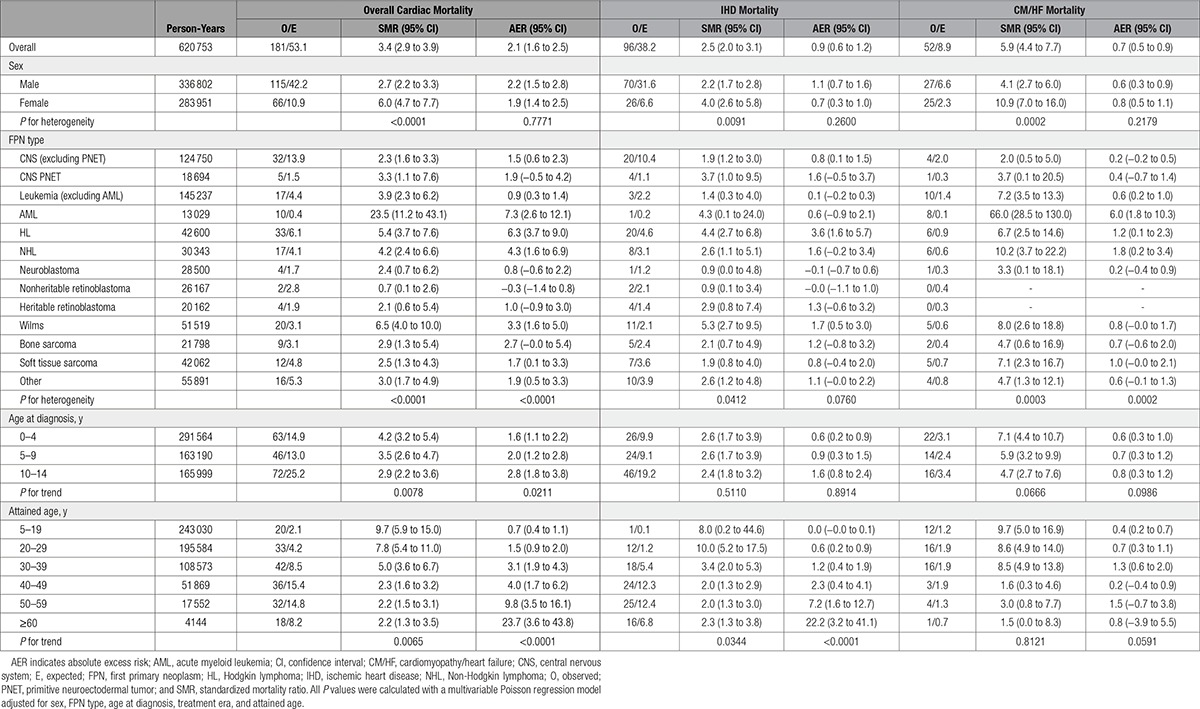
Standardized Mortality Ratios and Absolute Excess Risks per 10 000 Person-Years for Overall Cardiac Mortality, Ischemic Heart Disease Mortality, and Cardiomyopathy/Heart Failure Mortality, by Potential Explanatory Factors

**Table 4.**
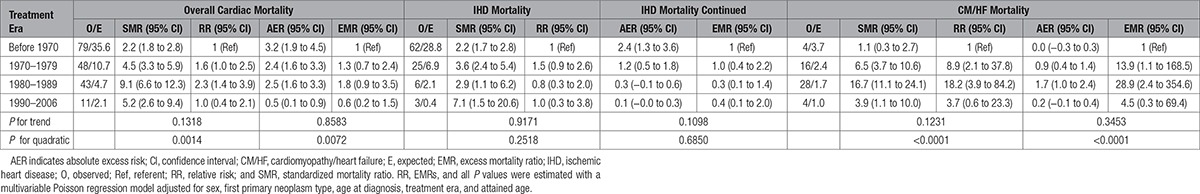
Standardized Mortality Ratios and Corresponding Relative Risks and Absolute Excess Risks per 10 000 Person-Years and Corresponding Excess Mortality Ratios for Each Treatment Era

In Table 5, we provide the percentage contributions of specific causes of death to the total excess number of deaths observed at different attained ages. Among those >60 years of age, cardiac deaths accounted for 22% and 41% of all excess deaths and all excess nonneoplastic deaths, respectively. Of all excess deaths observed among those >60 years of age, 31%, 22%, and 15% were attributable to subsequent primary neoplasms (SPNs), cardiac disease, and other circulatory diseases, respectively, accounting for 68% of the excess number of deaths overall.

**Table 5.**
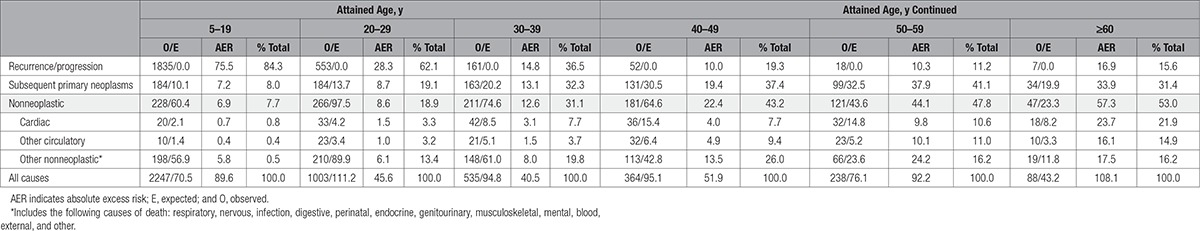
Absolute Excess Risks for Recurrences/Progression, Subsequent Primary Neoplasms, Nonneoplastic, Cardiac, Other Circulatory, and Other Nonneoplastic Causes of Death, by Attained Age as a Proportion of Total Excess Risk

**Figure 1.**
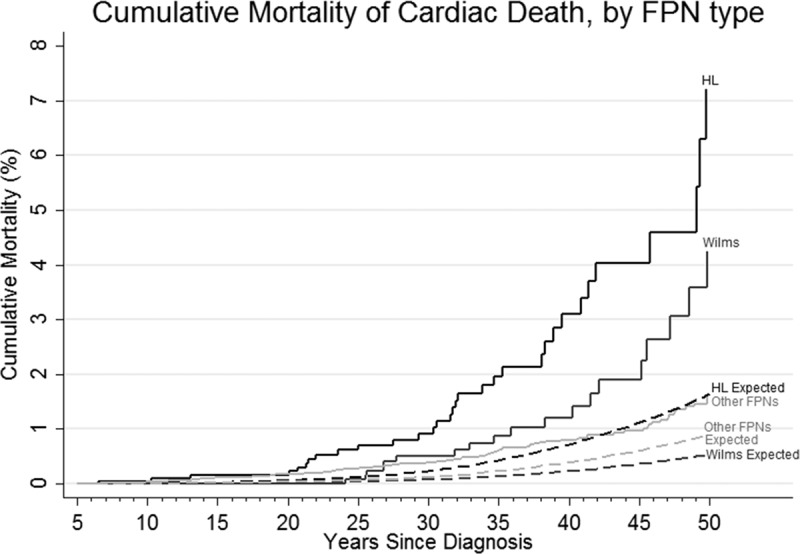
**Cumulative mortality for cardiac death by first primary neoplasm (FPN) diagnostic groups, by follow-up (years since diagnosis), compared with that expected.** HL indicates Hodgkin lymphoma.

Overall, the cumulative mortality for cardiac death was 5.0% at 60 years of follow-up compared with 3.5% expected (Figure I in the online-only Data Supplement). Among the FPN types, the cumulative mortality was greatest for survivors of Wilms and HL (Figure 1); the cumulative mortality for HL survivors began to increase at 20 years of follow-up compared with 25 years of follow-up in Wilms survivors and increased at a higher rate than that observed for Wilms survivors.

### Cardiac-Specific Mortality

Of the 181 cardiac deaths observed, there were 96 IHD, 52 CM/HF, 8 valvular disease, 5 arrhythmia, 2 pericardial disease, and 18 other cardiac deaths (7 pulmonary heart disease, 4 acute/subacute infective endocarditis, 4 cardiovascular disease unspecified, 1 hypertensive heart and renal disease, 1 hypertensive heart disease without heart failure, 1 other ill-defined heart disease; Table 2). The SMRs for IHD, CM/HF, valvular, arrhythmia, pericardial, and other cardiac deaths were as follows: 2.5 (95% CI, 2.0–3.1), 5.9 (95% CI, 4.4–7.7), 3.6 (95% CI, 1.6–7.2), 3.4 (95% CI, 1.1–8.0), 8.0 (95% CI, 1.0–29.0), and 8.3 (95% CI, 4.9–13.1), respectively. Because IHD and CM/HF accounted for >80% of cardiac deaths overall, we consider only these 2 specific cardiac outcomes separately.

### IHD Mortality

The cumulative mortality of IHD deaths increased steadily until ≈45 years of follow-up, at which point there was a steeper increase, ultimately reaching 3.8% at 65 years of follow-up, which was 1.0% higher than expected (Figure I in the online-only Data Supplement). The SMR for IHD death was highest for survivors of Wilms (SMR, 5.3; 95% CI, 2.7–9.5) and HL (SMR, 4.4; 95% CI, 2.7–6.8; Table 3). Survivors of central nervous system tumors (excluding primitive neuroectodermal tumor), non- Hodgkin lymphoma (NHL), and ‘other’ FPN types also had a statistically significant elevated risk of IHD death. As attained age increased, the SMR declined significantly (*P* for trend=0.0344) and AER increased significantly (*P* for trend<0.0001); beyond 60 years of age, survivors remained >2 times (SMR, 2.3; 95% CI, 1.3–3.8) more at risk than expected, which equated to 22.2 (95% CI, 3.2–41.1) excess IHD deaths per 10 000 person-years. There was no evidence of a relationship between treatment era and the excess risk (multiplicative or additive) of IHD deaths (Table 4).

### CM/HF Mortality

**Figure 2.**
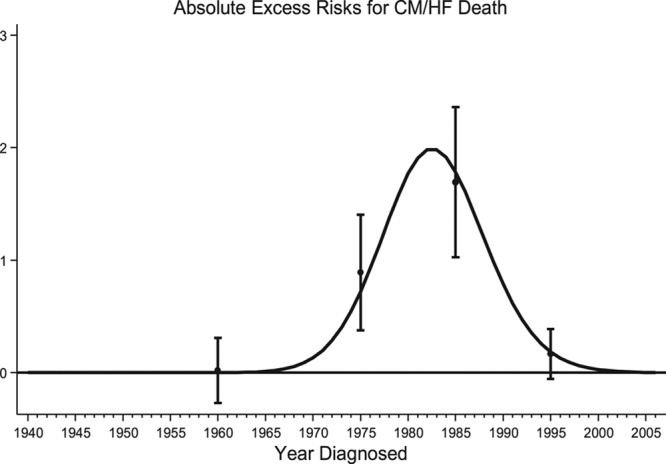
**Absolute excess risk per 10** **000 person-years for cardiomyopathy/heart failure (CM/HF) deaths for each calendar year of diagnosis.** The curve was produced by fitting a restricted cubic spline with the number of CM/HF deaths per 1 calendar year band as a weight. Point estimates of the absolute excess risk with corresponding 95% confidence intervals are plotted at the mean of the relevant treatment era (1940–1969, 1970–1979, 1980–1989, and 1990–2006).

At 65 years from diagnosis, the cumulative mortality of CM/HF deaths was 0.5% compared with 0.3% expected (Figure I in the online-only Data Supplement). All FPN types with at least 5 observed CM/HF deaths were found to be at a substantially higher risk than expected of CM/HF death (SMR>5.0; Table 3); survivors of acute myeloid leukemia, NHL, and Wilms were greatest at risk, with 66.0 times (95% CI, 28.5–130.0), 10.2 times (95% CI, 3.7–22.2), and 8.0 times (95% CI, 2.6–18.8) the number of expected CM/HF deaths, respectively. After adjustment, there was no evidence of a linear increase or decrease in excess risk of CM/HF death with attained age (SMR *P* for trend=0.8121, AER *P* for trend=0.0591). However, with regard to treatment era, evidence of a quadratic relationship was found for both the SMRs (*P* for quadratic<0.0001) and AERs (*P* for quadratic<0.0001; Table 4). More specifically, the number of excess CM/HF deaths increased with treatment era beginning around 1970, peaked among those treated in the 1980s, and then subsequently declined among those treated more recently (Figure 2). After adjustment for sex, FPN type, age at diagnosis, and attained age, survivors diagnosed from 1970 to 1979, 1980 to 1989, and 1990 to 2006 had 13.9 times (95% CI, 1.1–168.5), 28.9 times (95% CI, 2.4–354.6), and 4.5 times (95% CI, 0.3–69.4) more excess CM/HF deaths, respectively, than survivors diagnosed before 1970 (Table 4).

## Discussion

This largest ever study of cardiac mortality after childhood cancer within a cohort of 34 489 five-year survivors provides an unprecedented opportunity to investigate the impact of treatment era on the risk of cardiac death. Furthermore, with 73 565 person-years and 86 cardiac deaths among those >40 years of age and 21 696 person-years and 50 cardiac deaths among those >50 years of age, this is the first study to satisfactorily assess the pattern of excess cardiac deaths among survivors >50 years old. In doing so, this study adds 250 728 person-years and 76 cardiac deaths to our previous largest study assessing cardiac mortality.^14^ Furthermore, this study expands on and addresses many of the limitations of previous work assessing mortality among childhood cancer survivors from the United States^3,4,15^ and Nordic countries.^16,17^

### Principal Findings and Comparisons With Other Studies

Although 2 previous studies have assessed trends in the risk of cardiac causes of death among 5-year survivors of childhood cancer in relation to treatment era,^3,4^ this is the first study to provide evidence of a quadratic relationship for the excess risk of cardiac death overall and for CM/HF death in relation to treatment era, for which the number of excess deaths increases with treatment era, peaks among those treated in the 1980s, and then subsequently declines among those treated more recently. This relationship corresponds closely to the introduction of anthracycline chemotherapy, which has been shown to increase the risk of dilated cardiomyopathy^18–20^ and congestive heart failure.^21^ However, these results differ from a CCSS report^3^ that found a significant decline in the adjusted relative rate of death resulting from cardiac causes with more recent treatment era. Although the CCSS has a more restricted diagnosis period (1970–1999, 30 years) compared with our study (1940–2006, 66 years), it is unlikely that this explains the difference observed because we did not observe a linear decline in excess cardiac deaths with treatment era after restricting our diagnosis period to match the CCSS (SMR *P* for trend=0.7687, AER *P* for trend=0.3521; Table VI in the online-only Data Supplement). In fact, a quadratic relationship between treatment era and excess cardiac deaths was observed in the BCCSS when the diagnosis period was restricted to 1970 to 1999 (SMR *P* for quadratic=0.0024, AER *P* for quadratic=0.0019). A possible explanation for the difference observed between the BCCSS and CCSS would be differences in childhood cancer treatment exposures related to the development of cardiac diseases between the 2 studies. However, it is not possible to explore this hypothesis because of the lack of detailed treatment information for the BCCSS.

Overall, cardiac mortality estimates in this cohort were elevated at 3 times that expected. The risk of cardiac death remained elevated beyond 60 years of age, and the number of excess cardiac deaths was observed to increase significantly with age. Among those >60 years of age, cardiac deaths accounted for 22% and 41% of all excess deaths and all nonneoplastic excess deaths, respectively. This finding provides evidence that as survivors age beyond 60 years, circulatory deaths account for more excess deaths, 37% (60% of which was due to cardiac causes), than SPNs, 31%. With 31%, 22%, and 15% (in total 68%) of all excess deaths among those >60 years of age attributable to SPNs, cardiac disease, and other circulatory conditions, respectively, there is a clear message in terms of prevention of premature morbidity and mortality: Specific interventions, in terms of surveillance and treatment, that reduce SPNs and circulatory disease in survivors of childhood cancer are needed.

Another finding worth noting from our study relates to the risk of cardiac death by FPN type. Although HL survivors have long been recognized as having an increased risk of cardiac death, leading much research to focus on these survivors,^22–24^ Wilms survivors were in fact found to have a greater risk of cardiac death than HL survivors. In particular, Wilms survivors were found to have the greatest risk of IHD death among all FPN types; this finding suggests that hypertension and having 1 kidney are strong risk factors in Wilms survivors for cardiac death because the cardiac radiotherapy exposure in these survivors would be expected to be much lower than that used for HL survivors.

### Special Consideration

Despite the increased risk of cardiac death reported in this study during the 1980s, it is important to recognize that many more children diagnosed with cancer in the 1980s have survived as a result of anthracycline chemotherapy than have died as a result of its late effects. Furthermore, the fact that the excess risk of cardiac death has subsequently declined in those diagnosed in the 1990s and 2000s suggests that measures to reduce cardiotoxicity through the use of alternative drugs, lower cumulative doses, and improved monitoring and intervention appears to be having a beneficial effect for those treated more recently.

### Strengths and Weaknesses

The main strengths of our study are its large size, population-based design, wide diagnosis period, and long available follow-up time, which have provided an exceptional opportunity to assess cardiac mortality after childhood cancer. Through these strengths, we have been able to assess the impact of cancer treatment on cardiac mortality from prechemotherapy treatment eras to modern therapies and protocols. We have also been able to provide for the first time results on the risk of cardiac mortality among survivors of childhood cancer beyond 60 years of age. Because selection bias will be minimized through our population-based study design, these results are generalizable to survivors of childhood cancer in Great Britain, as well as other childhood cancer populations provided that they were treated with similar therapies. Therefore, the findings of this study provide useful evidence for survivors, clinicians, and clinical follow-up guidelines in Great Britain and potentially internationally.

A weakness of our study is the lack of detailed radiotherapy and chemotherapy information, which precluded any examination of dose-response patterns of treatment exposures in relation to cardiac mortality risk. However, the large-scale population-based design, wide spectrum of treatment eras, and very long follow-up available in this study provide opportunities not available in other cohorts. Another possible limitation of this study is that our classification of cardiac death relied on the underlying cause of death as listed on the death certificate, which has been previously shown to have imperfect accuracy.^25–28^ Although there is possible misclassification, it is more likely that we have underascertained cardiac deaths and thus underestimated the risk of cardiac death among childhood cancer survivors because these individuals are more likely to be coded as having a neoplastic-related death resulting from their previous medical history.^16^

### Reassessment of Findings

Over the treatment eras covered by this study, survival has improved substantially, and treatment regimens have changed markedly. Because of these evolving changes in survival and treatment exposures, reassessment of cardiac mortality after childhood cancer is crucial. For example, although we observed that the risk of cardiac death was greatest among those treated in the 1980s, it is important to determine whether with additional follow-up the more recently diagnosed survivors remain at a decreased risk or whether cardiac death has merely been delayed as a result of increased awareness, surveillance, and treatment of late effects after known cardiotoxic exposures. Similarly, we observed that cardiac death became one of the principal contributors to the excess number of deaths beyond 60 years of age. However, the survivors at least 60 years old at the time of our study are quite different from those more recently diagnosed in terms of both FPN types and treatment exposures. Therefore, a reassessment of the mortality risks in survivors >60 years of age is necessary in the future to determine the generalizability of our results for future childhood cancer survivors who reach 60 years of age.

### Future Research

At present, the Center for Childhood Cancer Survivor Studies is involved in several investigations aimed at improving knowledge of the risks and causes of cardiovascular complications among childhood cancer survivors. In particular, the center is collaborating in PanCareSurFup^29^ and PROCARDIO,^30^ both European consortiums investigating the link between cardiovascular disease and exposure of the heart and major vessels to radiotherapy and chemotherapy. For these projects, detailed treatment records were collected, and cumulative doses of radiation from radiotherapy and of each anticancer drug are being calculated. The resultant case-control studies will enable investigation of the dose-response between radiation and chemotherapy exposure of the heart and the risk of heart disease. The BCCSS cohort has also been linked to the national database of Hospital Episode Statistics, which is an electronic health record system for all hospitalizations in England. Through this linkage, the BCCSS will be in a stronger position to understand cardiac morbidity, and its treatment, in childhood cancer survivors. As a result of such initiatives, the BCCSS will be able to provide further insight into cardiovascular late effects among childhood cancer survivors in the United Kingdom.

### Conclusions and Implications

The greatest excess risks for deaths from CM/HF were observed among those treated between 1980 and 1989, which suggests that initiatives to reduce cardiotoxicity among those treated more recently may be having a measurable impact. Among those >60 years of age, SPNs, cardiac disease, and other circulatory conditions accounted for 31%, 22%, and 15% of all excess deaths, respectively, providing clear focus for preventive interventions. These findings provide important information for survivors, clinicians, and clinical guidelines, particularly in relation to preventing excess morbidity and mortality in survivors >60 years of age.

## Sources of Funding

This work was supported by grant number C386/A10422 from Cancer Research UK; PanCareSurFup, European 7th Framework Programme. Oxford funding was provided by Cancer Research UK (grant C8225/A21133) and by core funding to the Clinical Trial Service Unit (from Cancer Research UK, Medical Research Council, British Heart Foundation) and by the British Heart Foundation Centre for Research Excellence (grant RE/13/1/30181). No funder had a role in the study design, collection/analysis/interpretation of the data, writing of the report, or in the decision to submit the article for publication.

## Disclosures

All authors report no conflicts of interest in relation to this work. This report is independent research, and the views expressed in this article are not necessarily those of the National Health Service, the National Institute for Health Research, or the Department of Health.

## Supplementary Material

## Clinical Perspective

### What Is New?

- We report for the first time risks of cardiac mortality among 5-year survivors of childhood cancer beyond 50 years of age.
- In particular, among those >60 years of age, subsequent primary neoplasms, cardiac disease, and other circulatory conditions accounted for 31%, 22%, and 15% of all excess deaths, respectively, providing clear focus for preventive interventions.
- The present study is also the first to show that the number of excess cardiac deaths, in particular cardiomyopathy/heart failure deaths, was greatest among those diagnosed in the 1980s.

### What Are the Clinical Implications?

- Our findings suggest that cardiac disease becomes increasingly important as childhood cancer survivors reach mature adulthood, suggesting that initiatives aimed at preventing excess mortality and morbidity in survivors >50 years of age should be focused on both subsequent primary neoplasm and cardiac disease prevention, surveillance, and management.
- Nonetheless, efforts to reduce cardiotoxicity among those treated more recently appear to be having a measurable impact because the number of excess cardiac deaths was greatest among survivors diagnosed in the 1980s.
