# Supplementary material for: Population-Based Long-Term Cardiac-Specific Mortality Among 34 489 Five-Year Survivors of Childhood Cancer in Great Britain
Source: Circulation. 2017 Mar 6;135(10):951–63. doi: 10.1161/CIRCULATIONAHA.116.024811 (PMC5338891; doi:10.1161/CIRCULATIONAHA.116.024811)
Supplement: Supplementary file 1 [file cir-135-951-s001.pdf]

SUPPLEMENTAL MATERIAL:

Population-based long-term cardiac-specific mortality among 34,489 five-year survivors of childhood cancer in Great Britain.

Miranda M Fidler; Raoul C Reulen; Katherine Henson; Julie Kelly; David Cutter; Gill A Levitt; Clare Frobisher; David L Winter; Michael M Hawkins

On behalf of the British Childhood Cancer Survivor Study (BCCSS) Steering Group

Acknowledgements: The British Childhood Cancer Survivor Study (BCCSS) is a national collaborative undertaking guided by a steering group that comprises Douglas Easton (chair), Michael Hawkins, Helen Jenkinson, Meriel Jenney, Raoul Reulen, Kathryn Pritchard-Jones, Elaine Sugden, Charles Stiller, Andrew Toogood, and Hamish Wallace. The BCCSS benefits from the contributions of the officers, centres, and individual members of the Children's Cancer and Leukaemia Group and the Regional Paediatric Cancer Registries. The BCCSS acknowledges the collaboration of the Office for National Statistics, the National Records of Scotland, the Welsh Cancer Intelligence and Surveillance Unit, the Health & Social Care Information Centre, the National Cancer Intelligence Network, and Public Health England. The BCCSS would not have been possible without the support of our funders: Cancer Research UK and the European Commission to whom we offer our profound thanks. The views expressed in this publication are those of the authors and not necessarily represent those of the funders or collaborators. Finally, thanks to all BCCSS staff who have given many years of dedicated work to bring the BCCSS to fruition.

Characteristic	No. Dead	%	No. Alive	%	P-value	Total No.	%
**Overall**	4475	13.0	30014	87.0		34489	100.0
**Sex**							
Male	2629	13.9	16310	86.1		18939	100.0
Female	1846	11.9	13704	88.1	<0.001	15550	100.0
**First Primary Neoplasm Type**							
CNS (excluding PNET)	1334	19.1	5636	80.9		6970	100.0
CNS PNET	340	28.4	858	71.6		1198	100.0
Leukemia (excluding AML)	1104	11.6	8398	88.4		9493	100.0
AML	82	8.4	899	91.6		981	100.0
Hodgkin Lymphoma	331	14.8	1903	85.2		2234	100.0
Non-Hodgkin Lymphoma	131	8.5	1418	91.5		1549	100.0
Neuroblastoma	144	9.4	1391	90.6		1535	100.0
Non-Heritable Retinoblastoma	31	3.1	975	96.9		1006	100.0
Heritable Retinoblastoma	138	18.4	612	81.6		750	100.0
Wilms	184	7.7	2204	92.3		1388	100.0
Bone Sarcoma	198	16.6	997	83.4		1195	100.0
Soft Tissue Sarcoma	253	11.8	1894	88.2		2147	100.0
Other	205	6.7	2838	93.3	<0.001	3043	100.0
**Age at Diagnosis**							
Mean	7.3		6.5			6.6	
0-4	1662	10.6	14035	89.4		15697	100.0
5-9	1351	14.6	7913	85.4		9264	100.0
10-14	1462	15.3	8066	84.7	<0.001	9528	100.0
**Treatment Era**							
1940-1969	1329	35.5	2417	64.5		3746	100.0
1970-1979	1247	23.2	4132	76.8		5379	100.0
1980-1989	942	13.2	6205	86.8		7147	100.0
1990-1999	703	7.0	9328	93.0		10031	100.0
2000-2006	254	3.1	7932	96.9	<0.001	8186	100.0
**Years from Diagnosis**							
Mean	17.0		23.9			23.0	
5-9 years	2054	37.9	3368	62.2		5422	100.0
10-19 years	1060	9.1	10561	90.9		11621	100.0
20-29 years	548	6.9	7352	93.1		7900	100.0
30-39 years	417	7.7	5026	92.3		5443	100.0
40-49 years	277	9.9	2529	90.1		2806	100.0
50-59 years	103	8.9	1052	91.1		1155	100.0
60+ years	16	11.3	126	88.7	<0.001	142	100.0
**Attained Age at Exit**							
Mean	24.3		30.4			29.6	
5-9 years	416	48.9	435	51.1		851	100.0
10-19 years	1831	21.7	6614	78.3		8445	100.0
20-29 years	1003	9.4	9730	90.7		10733	100.0
30-39 years	535	8.1	6083	91.9		6618	100.0
40-49 years	364	7.5	4486	92.5		4850	100.0
50-59 years	238	11.4	1859	88.7		2097	100.0
60+ years	88	9.8	807	90.2	<0.001	895	100.0

Supplemental Table 1: Cohort characteristics of the British Childhood Cancer Survivor Study.
Abbreviations: CNS – central nervous system, PNET – primitive neuroectodermal tumor, AML – acute myeloid leukemia

Characteristic	Treatment Era							
	1940-1969		1970-1979		1980-1989		1990-2006	
	N	%	N	%	N	%	N	%
**Overall**	3746	10.9	5379	15.6	7147	20.7	18217	52.8
**Sex**								
Male	2019	53.9	2963	55.1	3941	55.1	10016	55.0
Female	1727	46.1	2416	44.9	3206	44.9	8201	45.0
**First Primary Neoplasm Type**								
CNS (excluding PNET)	1059	28.3	1094	20.3	1185	16.6	3632	19.9
CNS PNET	167	4.5	174	3.2	236	3.3	621	3.4
Leukemia (excluding AML)	168	4.5	1554	28.9	2206	30.9	5565	30.6
AML	10	0.3	68	1.3	193	2.7	710	3.9
Hodgkin Lymphoma	297	7.9	456	8.5	495	6.9	986	5.4
Non-Hodgkin Lymphoma	179	4.8	206	3.8	403	5.6	761	4.2
Neuroblastoma	217	5.8	153	2.8	307	4.3	858	4.7
Non-Heritable Retinoblastoma	255	6.8	195	3.6	160	2.2	396	2.2
Heritable Retinoblastoma	269	7.2	128	2.4	127	1.8	226	1.2
Wilms	324	8.7	480	8.9	515	7.2	1069	5.9
Bone Sarcoma	154	4.1	179	3.3	267	3.7	595	3.3
Soft Tissue Sarcoma	310	8.3	302	5.6	453	6.3	1082	5.9
Other	337	9.0	390	7.3	600	8.4	1716	9.4
**Age at Diagnosis**								
0-4	1727	46.1	2326	43.2	3317	46.4	8327	45.7
5-9	931	24.9	1567	29.1	1864	26.1	4902	26.9
10-14	1088	29.0	1486	27.6	1966	27.5	4988	27.4
**Years from Diagnosis**								
Mean	41.3		33.3		26.7		14.7	
5-9	458	12.2	524	9.7	459	6.4	3981	21.9
10-19	214	5.7	319	5.9	338	4.7	10750	59.0
20-29	166	4.4	243	4.5	4005	56.0	3486	19,1
30-39	202	5.4	2896	53.8	2345	32.8	0	0
40-49	1409	37.6	1397	26.0	0	0	0	0
50-59	1155	30.8	0	0	0	0	0	0
60+	142	3.8	0	0	0	0	0	0
**Attained Age at Exit**								
Mean	47.8		40.0		33.3		21.3	
5-9	86	2.3	105	2.0	95	1.3	565	3.1
10-19	405	10.8	478	8.9	429	6.0	7133	39,2
20-29	219	5.9	314	5.8	1474	20.6	8726	47.9
30-39	193	5.2	902	16.8	3730	52.2	1793	9,8
40-49	605	16.2	2826	52.5	1419	19.9	0	0
50-59	1343	35.9	754	14.0	0	0	0	0
60+	895	23.9	0	0	0	0	0	0

Supplemental Table 2: Cohort characteristics of the British Childhood Cancer Survivor Study by treatment era.
Abbreviations: CNS – central nervous system, PNET – primitive neuroectodermal tumor, AML – acute myeloid leukemia

First Primary Neoplasm Type	Attained Age (years)													
	5-9		10-19		20-29		30-39		40-49		50-59		60+	
	N	%	N	%	N	%	N	%	N	%	N	%	N	%
CNS (excluding PNET)	159	18.7	1553	18.4	2258	21.0	1196	18.1	977	20.1	559	26.7	268	29.9
CNS PNET	19	2.2	371	4.4	381	3.6	193	2.9	148	3.1	62	3.0	24	2.3
Leukemia (excluding AML)	285	33.5	2987	35.4	3092	28.8	1892	28.6	1102	22.7	126	6.0	9	1.0
AML	36	4.2	320	3.8	363	3.4	181	2.7	58	1.2	22	1.1	1	0.1
Hodgkin Lymphoma	3	0.4	248	2.9	705	6.6	471	7.1	497	10.3	228	10.9	82	9.2
Non-Hodgkin Lymphoma	14	1.7	217	2.6	457	4.3	417	6.3	255	5.3	123	5.9	66	7.4
Neuroblastoma	100	11.8	550	6.5	412	3.8	225	3.4	135	2.8	95	4.5	18	2.0
Non-Heritable Retinoblastoma	31	3.6	214	2.5	230	2.1	154	2.3	201	4.1	122	5.8	54	6.0
Heritable Retinoblastoma	51	6.0	151	1.8	148	1.4	127	1.9	142	2.9	97	4.6	34	3.8
Wilms	48	5.6	624	7.4	627	5.8	488	7.4	405	8.4	155	7.4	41	4.6
Bone Sarcoma	4	0.5	182	2.2	365	3.4	270	4.1	203	4.2	100	4.8	71	7.9
Soft Tissue Sarcoma	35	4.1	454	5.4	676	6.3	409	6.2	306	6.3	173	8.3	94	10.5
Other	66	7.8	574	6.8	1019	9.5	595	9.0	421	8.7	235	11.2	133	14.9

Supplemental Table 3: First primary neoplasm diagnoses of the British Childhood Cancer Survivor Study by attained age.

Abbreviations: CNS – central nervous system, PNET – primitive neuroectodermal tumor, AML – acute myeloid leukemia

	International Classification of Diseases (ICD) revision (calendar years of death)					
**Cause of Death**	**ICD-5 (1940-49)**	**ICD-6 (1950-57)**	**ICD-7 (1958-67)**	**ICD-8 (1968-78)**	**ICD-9 (1979-2000)**	**ICD-10 (2001-09)**
**All Cardiac**	58a-c, 90a-b, 91a-c, 92-95, 111b-c, 199, 200a(1)	401, 402.1, 410-416, 420-422, 430-434, 440-443	401, 402.1, 410-416, 420-422, 430-434, 440-443	391, 392.0, 393-398, 402, 404, 410-414, 420-429	391, 392.0, 393-398, 402, 404, 410-414, 416.1-9, 420-429	I01, I02.0, I05-I09, I11, I13, I20-I25, I27.1-9, I30-I52
Cardiomyopathy/ Heart Failure	58c, 93a-b, 93c(2-3), 93d, 111b-c, 200a(1)	401.2, 415, 422.0, 422.2, 431, 434.1-2	401.2, 415, 422.0, 422.2, 431, 434.1-2	391.2, 422, 425, 427.0, 427.1, 428	391.2, 398.0, 422, 425, 428, 429.0, 429.1, 429.3	I01.2, I09.0, I11.0, I13.0, I13.2, I40-I43, I50, I51.4-5, I51.7
Valvular Heart Disease	92a, 92b(1), 92c	421	421	394.9, 395.9, 396.9, 397.0, 424	424	I34-I39
Rheumatic Valvular Heart Disease	58b, 92b(2)	401.1, 410-414	401.1, 410-414	391.1, 394.0, 395.0, 396.0, 397.9	391.1, 394-397	I01.1, I05-I08, I09.1
Ischemic Heart Disease	93c(1), 94a, 94b	420, 422.1	420, 422.1	410-414	410-414, 429.7	I20-I25
Arrhythmias	199	433.0-1	433.0-1	427.2-9	426-427	I44-49
Pericardial Disease	58a, 90a-b	401.0, 432	401.0, 432	391.0, 393, 420, 423	391.0, 393, 420, 423	I01.0, I09.2, I30-I32
Other Cardiac	91a-c, 95	401.3, 402.1, 416, 430, 433.2, 434.0, 434.3, 440-443	401.3, 402.1, 416, 430, 433.2, 434.0, 434.3-4, 440-443	391.9, 392.0, 398, 402, 404, 421, 426, 429	391.8-9, 392.0, 389.9, 402, 404, 416.1-9, 421, 429.2, 429.4-6, 429.8-9	I01.8-9, I02.0, 109.8-9, I11.9, I13.1, I13.9, I27.1-9, I33, I51.0-3, I51.6, I51.8-9, I52

Supplemental Table 4: International Classification of Diseases (ICD) categorizations and sub-categorizations for cardiac causes of death as used in the analysis.

	Overall Cardiac Mortality		Ischemic Heart Disease Mortality		Cardiomyopathy/Heart Failure Mortality	
	**RR (95%CI)**	**EMR (95%CI)**	**RR (95%CI)**	**EMR (95%CI)**	**RR (95%CI)**	**EMR (95%CI)**
**Sex**						
Male	1 (ref)	1 (ref)	1 (ref)	1 (ref)	1 (ref)	1 (ref)
Female	2.2 (1.6,3.0)	1.1 (0.7,1.6)	1.9 (1.2,3.0)	0.7 (0.4,1.3)	3.0 (1.7,5.2)	1.5 (0.8,2.9)
Pheterogeneity	<0.0001	0.7771	0.0091	0.2600	0.0002	0.2179
**First Primary Neoplasm Type**						
CNS (excluding PNET)	1 (ref)	1 (ref)	1 (ref)	1 (ref)	1 (ref)	1 (ref)
CNS PNET	1.2 (0.5,3.2)	0.9 (0.2,5.4)	1.9 (0.6,5.5)	1.7 (0.3,12.5)	1.7 (0.2,15.0)	3.8 (0.2,61.0)
Leukemia (excluding AML)	0.7 (0.4,1.4)	0.8 (0.3,1.8)	0.4 (0.1,1.5)	0.2 (0.0,4.4)	1.7 (0.5,5.4)	3.2 (0.4,23.6)
AML	5.8 (2.8,12.1)	7.6 (3.2,18.1)	1.7 (0.2,12.6)	2.3 (0.2,23.5)	19.2 (5.7,64.8)	39.8 (5.5,289.6)
Hodgkin Lymphoma	2.7 (1.6,4.4)	4.0 (1.9,8.2)	2.4 (1.3,4.5)	3.6 (1.3,9.8)	3.8 (1.0,13.6)	4.9 (0.5,45.8)
Non-Hodgkin Lymphoma	2.0 (1.1,3.6)	3.0 (1.4,6.6)	1.4 (0.6,3.3)	2.1 (0.6,7.1)	5.4 (1.5,19.2)	13.8 (1.8,106.1)
Neuroblastoma	0.7 (0.2,2.1)	0.8 (0.2,3.0)	0.4 (0.1,3.2)	0.5 (0.0,9.1)	1.2 (0.1,10.7)	2.2 (0.1,38.4)
Non-Heritable Retinoblastoma	0.2 (0.1,1.0)	0.1 (0.0,3.2)	0.5 (0.1,2.1)	0.5 (0.0,5.0)	*NE*	*NE*
Heritable Retinoblastoma	0.7 (0.2,2.0)	0.4 (0.0,4.7)	1.4 (0.4,4.5)	1.5 (0.2,9.9)	*NE*	*NE*
Wilms	1.7 (0.9,3.1)	1.9 (0.8,4.3)	2.2 (1.0,5.1)	2.8 (0.8,9.5)	2.5 (0.7,9.9)	5.0 (0.6,41.7)
Bone Sarcoma	1.4 (0.6,2.9)	1.6 (0.5,4.9)	1.1 (0.4,3.0)	0.9 (0.1,8.6)	2.6 (0.5,14.2)	6.2 (0.5,71.1)
Soft Tissue Sarcoma	1.1 (0.5,2.1)	1.0 (0.3,2.9)	1.0 (0.4,2.4)	0.9 (0.2,4.5)	3.5 (0.9,13.2)	8.0 (1.0,65.0)
Other	1.1 (0.6,2.1)	1.0 (0.4,2.8)	1.2 (0.6,2.6)	1.2 (0.3,4.6)	2.1 (0.5,8.3)	3.6 (0.4,36.0)
Pheterogeneity	<0.0001	<0.0001	0.0412	0.0760	0.0003	0.0002
**Age at Diagnosis**						
0-4 years	1 (ref)	1 (ref)	1 (ref)	1 (ref)	1 (ref)	1 (ref)
5-9 years	0.7 (0.5,1.1)	0.7 (0.4,1.2)	0.9 (0.5,1.8)	1.0 (0.4,2.8)	0.7 (0.3,1.4)	0.7 (0.3,1.6)
10-14 years	0.5 (0.4,0.8)	0.5 (0.3,0.9)	0.8 (0.4,1.6)	1.0 (0.3,2.7)	0.5 (0.2,1.1)	0.5 (0.2,1.2)
Ptrend	0.0078	0.0211	0.5110	0.8914	0.0666	0.0986
**Treatment Era**						
<1970	1 (ref)	1 (ref)	1 (ref)	1 (ref)	1 (ref)	1 (ref)
1970-1979	1.6 (1.0,2.5)	1.3 (0.7,2.4)	1.5 (0.9,2.6)	1.0 (0.4,2.2)	8.9 (2.1,37.8)	13.9 (1.1,168.5)
1980-1989	2.3 (1.4,3.9)	1.8 (0.9,3.5)	0.8 (0.3,2.0)	0.3 (0.1,1.4)	18.2 (3.9,84.2)	28.9 (2.4,354.6)
1990-2006	1.0 (0.4,2.1)	0.6 (0.2,1.5)	1.0 (0.3,3.8)	0.4 (0.1,2.0)	3.7 (0.6,23.3)	4.5 (0.3,69.4)
Ptrend	0.1318	0.8583	0.9171	0.1098	0.1231	0.3453
Pquadratic	0.0014	0.0072	0.2518	0.6850	<0.0001	<0.0001
**Attained Age**						
5-19 years	1 (ref)	1 (ref)	1 (ref)	1 (ref)	1 (ref)	1 (ref)
20-29 years	0.8 (0.5,1.5)	1.9 (1.0,3.7)	1.3 (0.2,10.0)	11.4 (1.3,102.2)	0.9 (0.4,2.0)	1.6 (0.7,3.6)
30-39 years	0.6 (0.3,1.0)	3.6 (1.8,7.2)	0.4 (0.0,3.1)	19.8 (2.2,182.6)	1.0 (0.4,2.3)	2.9 (1.2,7.2)
40-49 years	0.3 (0.2,0.7)	5.7 (2.5,13.0)	0.2 (0.0,1.8)	30.1 (3.0,298.5)	0.4 (0.1,1.4)	*NE*
50-59 years	0.4 (0.2,0.8)	17.1 (6.6,44.7)	0.2 (0.0,2.0)	93.2 (9.1,949.8)	2.2 (0.5,10.4)	21.3 (3.1,147.8)
60+ years	0.4 (0.2,1.0)	45.7 (14.5,144.1)	0.3 (0.0,2.6)	267.5 (23.9,2992.9)	2.0 (0.1,26.7)	*NE*
Ptrend	0.0065	<0.0001	0.0344	<0.0001	0.8121	0.0591

Supplemental Table 5: Relative risks and excess mortality ratios per 10,000 person-years estimated using a multivariable model, adjusting for sex, first primary neoplasm type, age at diagnosis, treatment era, and attained age.

Abbreviations: CNS – central nervous system, PNET – primitive neuroectodermal tumor, AML – acute myeloid leukemia, RR – relative risks, EMR – excess mortality ratios, CI – confidence intervals, NE – not estimable

	Overall Cardiac Mortality	
	**RR (95%CI)**	**EMR (95%CI)**
**Sex**		
Male	1 (ref)	1 (ref)
Female	2.4 (1.6,3.7)	1.1 (0.7,1.9)
Pheterogeneity	<0.0001	0.5805
**First Primary Neoplasm Diagnosis**		
CNS (excluding PNET)	1 (ref)	1 (ref)
CNS PNET	0.4 (0.1,2.9)	0.4 (0.0,6.6)
Leukemia (excluding AML)	0.6 (0.3,1.2)	0.7 (0.3,1.7)
AML	4.9 (2.2,11.1)	6.7 (2.5,17.9)
Hodgkin Lymphoma	2.2 (1.1,4.4)	3.3 (1.3,8.3)
Non-Hodgkin Lymphoma	2.0 (0.9,4.5)	3.1 (1.2,8.4)
Neuroblastoma	0.3 (0.0,2.4)	0.4 (0.0,3.6)
Non-Heritable Retinoblastoma	0.3 (0.0,2.6)	0.4 (0.0,4.7)
Heritable Retinoblastoma	*NA*	*NA*
Wilms	1.2 (0.5,2.7)	1.4 (0.5,3.9)
Bone Sarcoma	1.8 (0.7,4.6)	2.5 (0.7,8.4)
Soft Tissue Sarcoma	1.2 (0.5,3.0)	1.6 (0.5,4.8)
Other	0.8 (0.3,2.0)	0.7 (0.2,2.9)
Pheterogeneity	0.0002	0.0001
**Age at Diagnosis**		
0-4 years	1 (ref)	1 (ref)
5-9 years	0.6 (0.4,1.1)	0.7 (0.4,1.2)
10-14 years	0.5 (0.3,0.9)	0.5 (0.2,0.9)
Ptrend	0.0216	0.0312
**Treatment Era**		
1970-1974	1 (ref)	1 (ref)
1975-1979	1.6 (0.9,2.9)	1.8 (0.8,4.2)
1980-1984	1.9 (1.0,3.5)	2.1 (0.9,4.8)
1985-1989	1.8 (0.9,3.7)	1.9 (0.7,4.6)
1990-1994	0.9 (0.4,2.4)	0.8 (0.3,2.7)
1995-1999	0.6 (0.2,2.1)	0.4 (0.1,2.4)
Ptrend	0.7687	0.3521
Pquadratic	0.0024	0.0019
**Attained Age**		
5-19 years	1 (ref)	1 (ref)
20-29 years	0.8 (0.4,1.4)	1.8 (0.9,3.7)
30-39 years	0.5 (0.2,0.9)	3.0 (1.4,6.3)
40-49 years	0.3 (0.2,0.8)	4.9 (1.8,13.5)
50+ years	0.3 (0.1,1.4)	15.8 (2.5,101.2)
Ptrend	0.0068	0.0004

Supplemental Table 6: Relative risks and excess mortality ratios per 10,000 person-years estimated using a multivariable model, adjusting for sex, first primary neoplasm type, age at diagnosis, treatment era, and attained age. For these analyses, the British Childhood Cancer Survivor Study was restricted to the diagnosis period to 1970-1999 in order to compare with the Childhood Cancer Survivor Study.

Abbreviations: CNS – central nervous system, PNET – primitive neuroectodermal tumor, AML – acute myeloid leukemia, RR – relative risks, EMR – excess mortality ratios, CI – confidence interval, NA – not applicable

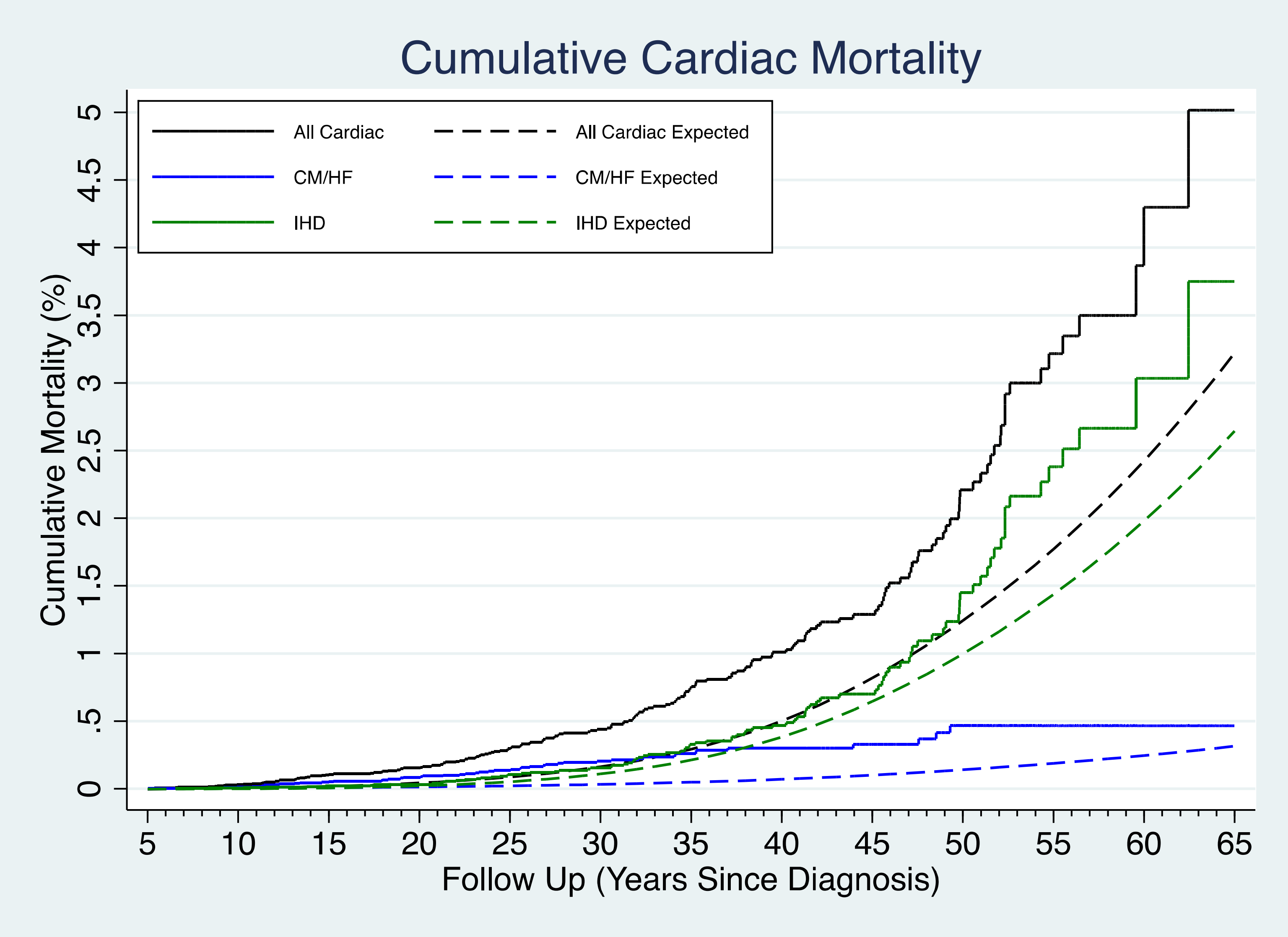

Supplemental Figure 1: Cumulative mortality of all cardiac causes, ischemic heart disease causes, and cardiomyopathy/heart failure causes among childhood cancer survivors compared to that expected in the general population, by follow-up (years since diagnosis).

Abbreviations: CM/HF – cardiomyopathy/heart failure, IHD – ischemic heart disease
